# Expression of key genes affecting artemisinin content in five *Artemisia* species

**DOI:** 10.1038/s41598-018-31079-0

**Published:** 2018-08-23

**Authors:** Maryam Salehi, Ghasem Karimzadeh, Mohammad Reza Naghavi, Hassanali Naghdi Badi, Sajad Rashidi Monfared

**Affiliations:** 1 Department of Plant Genetics and Breeding, Faculty of Agriculture, Tarbiat Modares University, Tehran, P. O. Box 14115-336, Iran; 2 Agronomy and Plant Breeding Department, Agricultural College, University of Tehran, Karaj, Iran; 3 Medicinal Plants Research Center, Institute of Medicinal Plants, ACECR, Karaj, Iran; 4 Department of Agricultural Biotechnology, Faculty of Agriculture, Tarbiat Modares University, Tehran, Iran

## Abstract

Artemisinin, an effective anti-malarial drug is synthesized in the specialized 10-celled biseriate glandular trichomes of some *Artemisia* species. In order to have an insight into artemisinin biosynthesis in species other than *A*. *annua*, five species with different artemisinin contents were investigated for the expression of key genes that influence artemisinin content. The least relative expression of the examined terpene synthase genes accompanied with very low glandular trichome density (4 No. mm^−2^) and absence of artemisinin content in *A*. *khorassanica* (S2) underscored the vast metabolic capacity of glandular trichomes. *A*. *deserti* (S4) with artemisinin content of 5.13 mg g^−1^ DW had a very high expression of *Aa*-*ALDH1* and *Aa*-*CYP71AV1* and low expression of *Aa*-*DBR2*. It is possible to develop plants with high artemisinin synthesis ability by downregulating *Aa*-*ORA* in S4, which may result in the reduction of *Aa*-*ALDH1* and *Aa*-*CYP71AV1* genes expression and effectively change the metabolic flux to favor more of artemisinin production than artemisinic acid. Based on the results, the *Aa*-*ABCG6* transporter may be involved in trichome development. S4 had high transcript levels and larger glandular trichomes (3.46 fold) than *A*. *annua* found in Iran (S1), which may be due to the presence of more 2C-DNA (3.48 fold) in S4 than S1.

## Introduction

**Figure 1:**
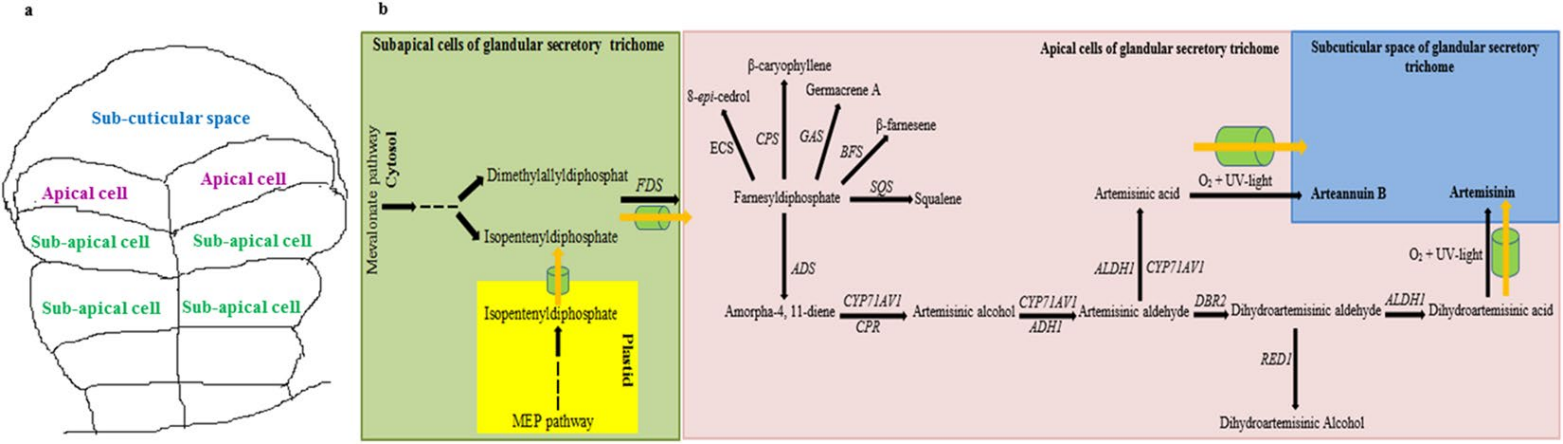
The specialized 10-celled biseriate glandular secretory trichome of *Artemisia annua* (**a**) and Summary of terpene metabolism and Transporters (ABCG6, ABCG7) involved in artemisinin biosynthesis and cuticle development (illustrated as green cylinders, (**b**)). ECS: epi-cedrol synthase, CPS: β-caryophyllene synthase, GAS: germacrene A synthase, BFS: β-farnesene synthase, SQS: Squalene synthase, FDS: farnesyl diphosphate synthase, ADS: amorpha-4,11-diene synthase, CYP71AV1: amorphadiene-12-hydroxylase, CPR: cytochrome P450 reductase, ADH1: alcohol dehydrogenase 1, ALDH1: aldehyde dehydrogenase 1, DBR2: artemisinic aldehyde Δ11(13) reductase, RED1: dihydroartemisinic aldehyde reductase.

The specialized 10-celled biseriate glandular trichomes (Fig. 1a) of some *Artemisia* species are the sites of artemisinin synthesis^1–6^. Artemisinin is a sesquiterpene lactone, an efficacious anti-malarial drug against a number of cancers and viral diseases^7^. *Artemisia* genus of Asteraceae family possesses over 500 species, which are mainly found in Asia, Europe and North America^8^. 35 of these species are found in Iran^9^. All *Artemisia* species produce less artemisinin contents than *A*. *annua*^1–6^. The main source of artemisinin is *A*. *annua*. Artemisinin yield by the wide type of *A*. *annua* is very low and insufficient to cover the need of all patients^10^.

Hitherto, metabolic engineering for high artemisinin production has failed due to lack of genetic evidence for the biosynthesis pathway^11^. The knowledge of factors influencing the entire biosynthetic pathway and mechanisms regulating the onset and flux of the pathway in other *Artemisia* species can lead to favorable metabolic engineering when compared to *A*. *annua*.

Artemisinin biosynthetic pathway in eight *Artemisia* species was studied. It was reported that *A*. *absinthium* had a higher expression level of both *Aa*-*ALDH1* and *Aa*-*CYP71AV1* genes when compared to *A*. *annua* during the developmental stages^5^. Salehi *et al*.^6^ investigated artemisinin biosynthetic pathway and two trichome formation genes in five *Artemisia* species. In addition to the genes that regulate trichome formation and the artemisinin pathway, artemisinin yield was affected by genes of branching pathways (Fig. 1b), transcription factors and transporters (Fig. 1b) involved in artemisinin biosynthesis. To the best of our knowledge, there are no published studies on sesquiterpene synthases (Fig. 1b) that compete for the same substrate, farnesyl diphosphate (FDP), transcription factors (TFs) and transporters (Fig. 1b) that are involved in artemisinin production in any other *Artemisia* species when compared to *A*. *annua*.

The basic C_5_ precursors for terpenoid biosynthesis (isopentenyl diphosphate (IDP) and dimethylallyl diphosphate (DMADP)) are synthesized via two distinct pathways: the mevalonate (MVA) pathway in the cytosol and the methylerythritol phosphate (MEP) pathway in plastid^12^. One molecule of IDP and one molecule of DMADP are condensed to produce geranyl diphosphate (GDP). GDP condenses with one unit of IDP to produce FDP. FDP is a significant product at the branching point of terpenoid metabolism. It is converted to β-farnesene, β-caryophyllene, germacrene A, *epi*-cedrol, squalene and amorphadiene by β-farnesene synthase (Aa-BFS), β-caryophyllene synthase (Aa-CPS), germacrene A synthase (Aa-GAS), *epi*-cedrol synthase (Aa-ECS), squalene synthase (Aa-SQS) and amorpha-4, 11-diene synthase (Aa-ADS), respectively^13–18^ (Fig. 1b). These products are produced in T-shaped trichomes or glandular trichomes or in both types of trichomes. Amorphadiene is first converted into dihydroartemisinic acid (DHAA) by a series of enzymes^19–22^ (Fig. 1b), and thereafter, DHAA is converted into artemisinin by an enzyme-independent reaction^23^. The expression of several sesquiterpene synthase genes and *Aa*-*SQS* may have a negative impact on artemisinin production in plants by competing for the same substrate, FDP^24^. Blocking of the branch pathways in *A*. *annua* is a useful technique for obtaining a high artemisinin producing plant^25^.

In plants, spatial-temporal regulation of secondary metabolites production and storage is usually regulated by TFs^26^. Overexpression of these factors have been proposed as an auspicious approach for increasing secondary metabolism in plants more efficiently since the plant transcription factors regulate a series of genes in one specific pathway^27^.

Artemisinin intermediates, especially the aldehydes are toxic to cells^28^. Some transporters exist between plastid and cytosol (transport of isopentenyl diphosphate from plastid to cytosol), subapical and apical cells (transport of FDP from subapical cells to apical cells), apical cells and subcuticular space (transport of artemisinin and arteannuin B from apical cells to subcuticular space) of glandular trichomes (Fig. 1b). These transporters carry the precursors to the sites where artemisinin is produced and accumulated^29^. Moreover, some transporters, which are involved in trichome development could affect artemisinin yield^29^.

Genome size (i.e. the DNA content of the unreplicated nucleus, 2C^30^; which is expressed in picograms or in millions base pairs, 1 pg = 978 Mbp^31^) as an important character in biodiversity correlates with many different kinds of biological parameters^32^. ‘C value’ (holoploid genome size) shows the DNA content of the unreplicated haploid complement irrespective of the degree of generative polyploidy, aneuploidies etc. ‘Monoploid genome size’ (1Cx) is the DNA content in a basic chromosome set (x) of a somatic cell^30^.

In the current study, five *Artemisia* species with different artemisinin contents were assessed in terms of expression of six terpene synthase genes (Fig. 1b) competing for the same substrate, FDP, three transcription factor genes (*Aa*-*ORA*, *Aa*-*ERF1*, *Aa*-*WIRKY1*) and two transporter genes (Fig. 1b) influencing artemisinin production. *A*. *deserti* (S4) had high transcript levels and was reported to have larger trichomes than *A*. *annua* found in Iran (S1, 3.46 fold)^6^. Consequently, the chromosome number and nuclear DNA content were also determined to identify the relationship of high transcript levels and gland size of S4 with its genome size.

## Results and Discussion

### Artemisinin content and glandular trichome density

**Figure 2:**
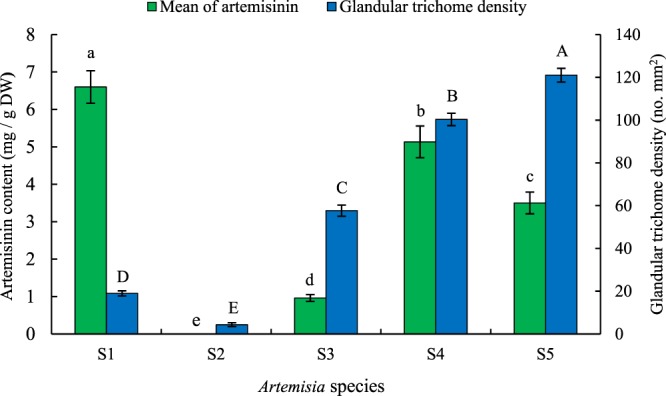
Artemisinin content and glandular trichome density of five *Artemisia* species including S1 (*A*. *annua* found in Iran), S2 (*A*. *khorassanica*), S3 (*A*. *persica*), S4 (*A*. *deserti*), and S5 (*A*. *marschalliana*). Error bars are shown as SE (n = 3). Means followed by the same letter are not significantly different according to the LSD at 0.01 probability level.

**Figure 3:**
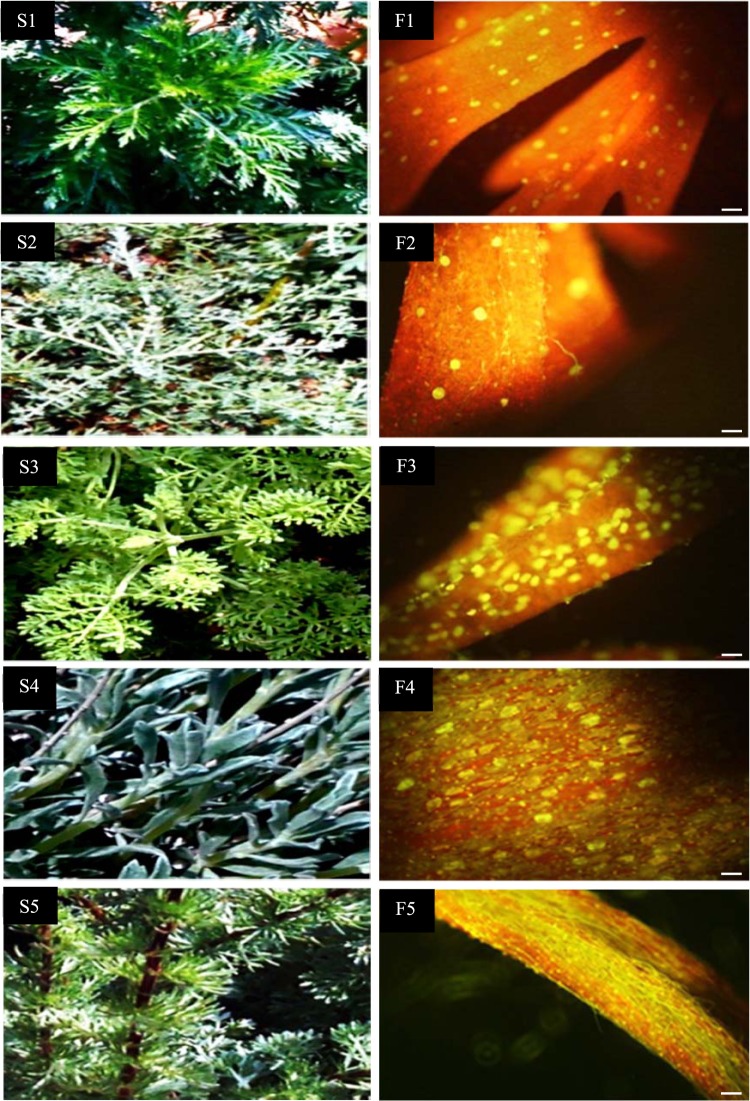
Glandular trichomes showing the content of autofluorescing aromatic oils (F1, F2, F3, F4, F5), Scale bar 100 μm, 10× objective, 10× on ocular of five *Artemisia* species including *A*. *annua* found in Iran (S1, F1), *A*. *khorassanica* (S2, F2), *A*. *persica* (S3, F3), *A*. *deserti* (S4, F4) and *A*. *marshalliana* (S5, F5).

S1 had the highest amount of artemisinin (6.60 mg g^−1^ DW) followed by S4 (5.13 mg g^−1^ DW), S5 (3.50 mg g^−1^ DW) and S3 (0.96 mg g^−1^ DW). No artemisinin content was observed in S2 (Fig. 2). All previous studies revealed that other *Artemisia* species produced less artemisinin compared to *A*. *annua*^1–6^. *A*. *annua* L. found in Iran has been reported to be a low artemisinin producing plant^6,33^. In the present study, S4 had slightly less artemisinin content than S1 (Fig. 2). The glandular trichome densities of five *Artemisia* species were determined employing fluorescence microscopy images (Fig. 3). The highest glandular trichome density was observed in S5 (121 No. mm^−2^) followed by S4 (100 No. mm^−2^), S3 (58 No. mm^−2^), S1 (19 No. mm^−2^) and S2 (4 No. mm^−2^, Fig. 2). Artemisinin content had no significant correlation (r = 0.25^ns^) with glandular trichome density.

### Gene expression

The qPCR technique was applied to ascertain the relationship of artemisinin content with the expression pattern of key genes influencing artemisinin content.

### Relative expression analysis of six terpene synthase genes

**Figure 4:**
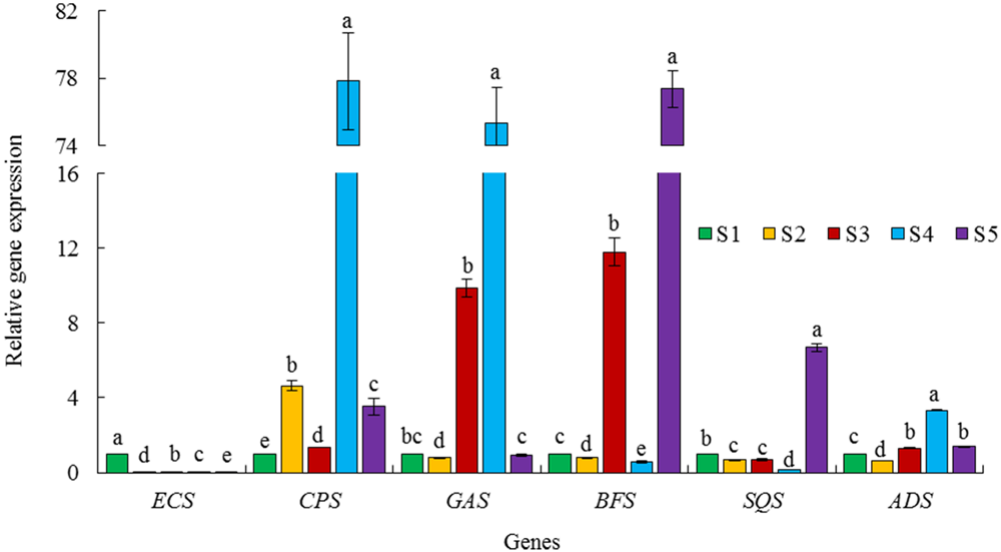
Relative expression of six terpene synthase genes in five *Artemisia* species including S1 (*A*. *annua* found in Iran), S2 (*A*. *khorassanica*), S3 (*A*. *persica*), S4 (*A*. *deserti*), and S5 (*A*. *marschalliana*). Error bars are shown as SE (n = 3). Means within a gene followed by the same letter are not significantly different according to the LSD at 0.01 probability level.

The sesquiterpene synthases including Aa-ADS (amorpha-4, 11-diene synthase), Aa-ECS (epi-cedrol synthase), Aa-CPS (β-caryophyllene synthase), Aa-GAS (germacrene A synthase), and Aa-BFS (β-farnesene synthase) compete for the same substrate, FDP (Fig. 1-b). In addition, FDP is used for the biosynthesis of sterols and triterpenes by squalene synthase (Aa-SQS, Fig. 1-b). Therefore, they may influence artemisinin production in a plant. S4 had higher expression of *Aa*-*ADS* (3.33 fold), *Aa*-*CPS* (77.81 fold) and *Aa*-*GAS* (75.29 fold) than S1 (Fig. 4). It can be concluded that the blocking of two genes (*Aa*-*CPS* and *Aa*-*GAS*) at the branching points in S4 may be an efficacious technique for inducing plants to produce higher levels of artemisinin. The high transcript levels of S4 may result in high metabolic capacity. However, the impact of post-transcriptional and translational regulation must also be considered. S5 had higher expression of *Aa*-*ADS* (1.39 fold), *Aa*-*BFS* (77.36 fold) and *Aa*-*SQS* (6.70 fold) than S1 (Fig. 4). S3 had relatively high glandular trichome density and very low artemisinin content (Fig. 2) due to higher expression of *Aa*-*CPS* (1.35 fold), *Aa*-*GAS* (9.87 fold) and *Aa*-*BFS* (11.78 fold, Fig. 4). S3 also had less expression of artemisinin biosynthesis genes compared to S1.

Artemisinin is synthesized in the specialized 10-celled glandular trichomes^34^. β-caryophyllene, a volatile metabolite is located in non-glandular trichomes^35^. β-farnesene, which plays an important role as an alarm pheromone^36,37^ is located in both glandular trichomes and non-glandular trichomes^35^. Aa-GAS is probably located in the glandular trichomes since it was cloned from glandular trichomes expressed sequence tag (EST) library^18^. Among six studied terpene synthase genes, only S2 had higher expression of *CPS* gene (4.64 fold) compared to S1 (Fig. 4). This is understandable because S2 had very low glandular trichome density (Fig. 2) and high non-glandular trichome density^6^. The least relative expression of the examined terpene synthase genes (Fig. 4) accompanied with very low glandular trichome density and absence of artemisinin content in S2 (Fig. 2) underscored the vast metabolic capacities of glandular trichomes.

While trichomes of many species produce a high content of one or a few specialized metabolites, it is possible that many others do not^38^. Trichomes function as a closed biochemical system with a simple input and little highly active biochemical pathways of both primary metabolism (for generating energy and precursors) and secondary (specialized) metabolism (for generating final products)^39^. Since the biosynthetic capacity of trichomes is limited by the amount and type of carbon source imported into them, it can be observed that the total output is limited when a given type of trichome is allowed to generate various classes of compounds^38^. Therefore, it can be concluded that blocking of active branch pathways in artemisinin producing plants is an effective technique for generating high yield artemisinin. The hairpin RNA-mediated gene silencing of *Aa*-*SQS* in *A*. *annua* resulted in downregulaton of *Aa*-SQS and 3-fold increase in artemisinin synthesis^40^. In addition, the blocking of branch pathways in *A*. *annua* was reported to be an efficacious method for generating high yield artemisinin^25^.

### Relative expression analysis of three transcription factors

**Figure 5:**
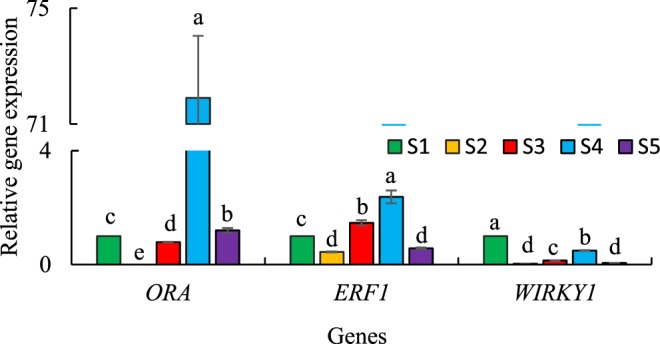
Relative expression of three transcription factor in five *Artemisia* species including S1 (*A*. *annua* found in Iran), S2 (*A*. *khorassanica*), S3 (*A*. *persica*), S4 (*A*. *deserti*), and S5 (*A*. *marschalliana*). Error bars are shown as SE (n = 3). Means within a gene followed by the same letter are not significantly different according to the LSD at 0.01 probability level.

Transcription factors regulate the activity of genes involved in the biosynthesis of secondary metabolites in plants by binding to the *cis*-acting regulatory elements of the promoters. Aa-WRKY1 had the ability to bind to the W-box in *Aa*-*ADS* promoter and activate *Aa*-*ADS* gene expression in transgenic tobacco plants and transient expression of *A*. *annua* leaf system^41^. In another study, overexpression of *Aa*-*WRKY* improved the transcription level of *Aa*-*CYP71AV1*, while the transcription levels of *Aa*-*ADS* and *Aa*-*DBR2* did not change significantly in transgenic plants^42^. The expression of *Aa*-*ORA*, *Aa*-*ADS*, *Aa*-*CYP71AV1* and *Aa*-*DBR2* were promoted in *Aa*-*ORA* overexpressing transgenic plants^43^. Aa-ERF1 was a positive regulator of *Aa*-*ADS* and *Aa*-*CYP71AV1*^44^. In this study, we monitored the relative expression of three transcription factors including *Aa*-*ORA*, *Aa*-*ERF1* and *Aa*-*WIRKY1* (Fig. 5). Gene expression of *Aa*-*ORA*, *Aa*-*ERF1* and *Aa*-*WIRKY1* in S4 were 71.90, 2.38 and 0.49 fold, respectively compared to S1 (Fig. 5). S3 and S5 had a little higher expression of *Aa*-*ERF1* (1.46 fold) and *Aa*-*ORA* (1.2 fold), respectively than S1 (Fig. 5). Since *Aa*-*ORA* (Fig. 5), *Aa*-*ALDH1* and *Aa*-*CYP* (data not shown) in the studied species were found to have a similar expression pattern, we suggest that *Aa*-*ORA* is a transcription factor that regulates the promoters of *Aa*-*ALDH1* and *Aa*-*CYP* genes. It may be concluded that S4 produced more artemisinic acid/arteannuin B than artemisinin because Aa-DBR2 and Aa-ALDH1 acted on the same pool of intermediates. The relative turnover of Aa-ALDH1 was much higher than Aa-DBR2 in S4^6^. Hence, the low artemisinin content of S4 was probably due to the flux of intermediate through the two branches (the oxidation of the artemisinic aldehyde to artemisinic acid or the reduction of the artemisinic aldehyde to dihydroartemisinic aldehyde, Fig. 1-b) of the pathway^6^. It is possible to develop high artemisinin producing plant by downregulating *Aa*-*ORA*, which may decrease the expression of *Aa*-*ALDH1* and *Aa*-*CYP* and change the metabolic flux more efficiently to artemisinin production than artemisinic acid.

### Relative expression analysis of *ABCG6* and *ABCG7* transporter unigenes

**Figure 6:**
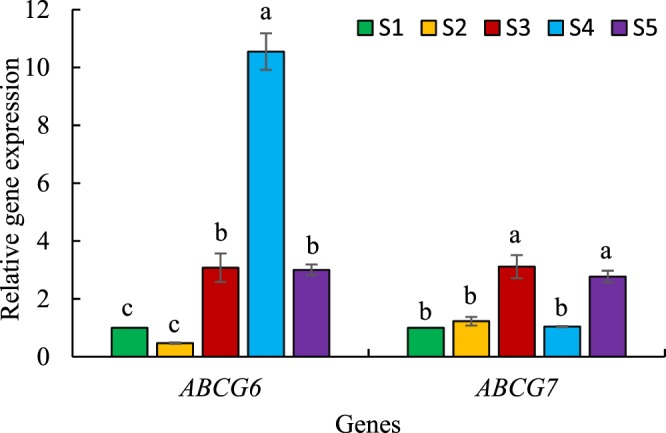
Relative expression of *ABCG6* and *ABCG7* transporter unigenes in five *Artemisia* species including S1 (*A*. *annua* found in Iran), S2 (*A*. *khorassanica*), S3 (*A*. *persica*), S4 (*A*. *deserti*), and S5 (*A*. *marschalliana*). Error bars are shown as SE (n = 3). Means within a gene followed by the same letter are not significantly different according to the LSD at 0.01 probability level.

Two ABC (ATP-binding cassette) transporter, *Aa*-*ABCG6* and *Aa*-*ABCG7* (Fig. 1-b) showed parallel expression pattern as two artemisinin biosynthesis specific genes (*Aa*-*ADS* and *Aa*-*CYP*)^29^. It was concluded that the two transporters were involved in *A*. *annua* glandular trichome cuticle formation and/or played roles in transportation that were related to artemisinin production and accumulation. We investigated genes expression of *Aa*-*ABCG6* and *Aa*-*ABCG7* as effective transporters in this study (Fig. 6). S3, S4 and S5 had higher glandular trichome density than S1 (Fig. 2), and these species also had higher expression of *Aa*-*ABCG6* gene (Fig. 6). The ABCG6 transporter most likely is not part of any transportation related to artemisinin production and accumulation because S3 with low artemisinin content had high expression of *ABCG6*. S2 had very low glandular trichome density (4 No. mm^−2^, Fig. 2) and less expression of *Aa*-*ABCG6* gene (0.57 fold, Fig. 6) than S1. Therefore, it can be speculated that the ABCG6 transporter might be involved in glandular trichome cuticle development. Some transporters involved in glandular trichome cuticle development could be relevant to artemisinin yield as previously reported^29^.

### Cytogenetic studies

**Figure 7:**
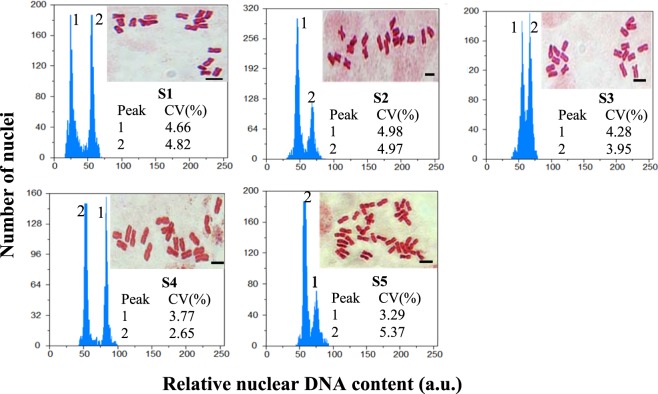
Histograms of flow cytometric 2C DNA content and somatic chromosomes of five *Artemisia* species including S1 (*A*. *annua* found in Iran), S2 (*A*. *khorassanica*), S3 (*A*. *persica*), S4 (*A*. *deserti*), and S5 (*A*. *marschalliana*).

Among five studied *Artemisia* species, four species (S1–S4) were diploid (2*n* = 2*x* = 18 m), while S5 was tetraploid (2*n* = 4*x* = 36 m, Fig. 7 and Table 1). In the diploid species, the mean CL was 4.52 μm, varying from 3.37 μm (S1) to 6.11 μm (S4, Supplementary Table S1) and the mean TCV was 7.04 μm^3^, ranging from 4.60 μm^3^ (S1) to 11.76 μm^3^ (S4, Supplementary Table S1). In the tetraploid species (S5), the mean CL and TCV were 4.35 μm and 5.92 μm^3^, respectively (Supplementary Table S1). The histograms used for analysis of the nuclear DNA content contained two peaks: peak 1 refers to the G1 of unknown *Artemisia* species samples and peak 2 represents the G1 of the known *Pisum sativum* cv. Ctirad (2C DNA = 9.09 pg) internal reference standard (Fig. 7). The variation coefficients (CV) of G1 peaks were less than 5% for *Artemisia* species and *P*. *sativum* samples. ANOVA showed significant differences between diploid (p < 0.01) species for nuclear 2C DNA amount. Interestingly, among the four diploid species, a difference of 9.97 pg in 2C value [4.02 (S1)–13.99 (S4)] was observed despite the four species having the same chromosome numbers of 18 (Table 1). Among the five studied *Artemisia* species, S1, which is an annual plant (4.05 pg) had the least 2C DNA content; S2-S5 are perennial plants. This is in consonance with the idea that a bigger genome implies a longer cell cycle; thus, they are prevented from the short life cycle that is typical of annual plants^45^. The diploid S4 with the most genome size, 2C DNA = 13.99 pg had the highest transcript levels.

**Table 1:** Mean (n = 3) 2C-DNA of five *Artemisia* species including S1 (*A*. *annua* found in Iran), S2 (*A*. *khorassanica*), S3 (*A*. *persica*), S4 (*A*. *deserti*), and S5 (*A*. *marschalliana*).

Species	Ploidy level	2n	2C DNA mean (pg) ± SE	Holoploid		Monoploid	
				(1C DNA) genome size (pg)	(1C DNA) genome size (Mbp)	(1Cx DNA) genome size (pg)	(1Cx DNA) genome size (Mbp)
S1	2*x*	18	4.02^d^ ± 0.02	2.010	1965.78	2.010	1965.78
S2	2*x*	18	6.12^c^ ± 0.05	3.060	2992.68	3.060	2992.68
S3	2*x*	18	7.14^b^ ± 0.08	3.570	3491.46	3.570	3491.46
S4	2*x*	18	13.99^a^ ± 0.06	6.995	6841.11	6.995	6841.11
S5	4*x*	36	11.50 ± 0.01	5.750	5623.50	2.875	2811.75

Means within a column followed by the same letter are not significantly different according to the LSD at the 0.01 probability level.

## Conclusion

Several *Artemisia* species produce less artemisinin than *A*. *annua*. The aim of our project was to comprehend the cause of the low artemisinin content of *Artemisia* species other than *A*. *annua* in order to have a better insight into artemisinin biosynthesis. Our previous study^6^ showed that biseriate, capitate glandular trichomes were prevalent in the genus of *Artemisia* and the density of glandular trichome and gland size had no significant relationship with artemisinin content. In addition, in our previous study^6^, the expression of artemisinin biosynthesis and trichome formation genes in five *Artemisia* species with different artemisinin content was reported.

In the current study, in order to have a better insight into artemisinin biosynthesis in *Artemisia* species other than *A*. *annua*, these five species with different artemisinin content were assessed in terms of key genes expression that affects artemisinin production including six terpene synthase genes (*Aa*-*ECS*, *Aa*-*CPS*, *Aa*-*GAS*, *Aa*-*BFS*, *Aa*-*ADS*, and *Aa*-*SQS*, Fig. 1-b), three transcription factor genes (*Aa*-*ORA*, *Aa*-*ERF1*, *Aa*-*WIRKY1*) and two transporter genes (*Aa*-*ABCG6* and *Aa*-*ABCG7*, Fig. 1-b).

S4 (*A*. *deserti*) had the highest expression of *Aa*-*ADS* (3.33 fold), *Aa*-*CPS* (77.81 fold) and *Aa*-*GAS* (75.29 fold, Fig. 4). Blocking of two genes at branching points (*Aa*-*CPS* and *Aa*-*GAS*) in S4 may be an efficacious method for generating high artemisinin producing plant.

*Aa*-*ORA*, *Aa*-*ALDH1* and *Aa*-*CYP* in the studied species had a similar expression pattern; we therefore suggested that *Aa*-*ORA* is a transcription factor that regulates the promoters of *Aa*-*ALDH1* and *Aa*-*CYP* genes. Our previous study^6^ showed that the relative turnover potential of ALDH1 was 112 folds higher than DBR2 in S4 (*A*. *deserti*) and converted more metabolic flux into artemisinic acid than artemisinin. It is possible to develop high artemisinin producing plant by the downregulation of *Aa*-*ORA*, which may decrease the expression of *Aa*-*ALDH1* and *Aa*-*CYP* and efficiently change more metabolic flux to favor artemisinin production compared to artemisinic acid.

The relationship between expression patterns of the two studied transporter genes (Fig. 6), artemisinin content and glandular trichome density (Fig. 2) in five *Artemisia* species suggested that *Aa*-*ABCG6* transporter plays a major role in glandular trichome development while it does not play a role in the transportation of artemisinin precursors.

There was a positive and significant correlation coefficient between individual data of monoploid genome size and area of glandular trichomes (r = 0.87**, Supplementary Fig. S1). The diploid S4 having the most genome size; 2C DNA = 13.99 pg (Table 1) had higher transcript levels and larger trichomes (3.46 fold, Supplementary Fig. S1) than *A*. *annua* found in Iran (S1). The amount of DNA control the cell size and cell cycle length such that the correlation of genome size and cell size was much stronger, more constant and direct than it was for cell cycle length^46^. It was reported that cell size correlated significantly with the transcription rate and that genome expression can be regulated genetically to induce changes in cell size^47^. The larger cells could sustain their larger biomass by owning more gene copies that can produce more RNAs and proteins accordingly^48^. Therfore, the high transcript levels of S4 might be due to the presence of more 2C value (3.48 times) in S4 than S1.

## Materials and Methods

Seeds of 17 *Artemisia* species were gathered from different parts of Iran^6^. Also, *A*. *annua* cv. Anamed which is regarded as a high artemisinin cultivar was included in the experiment^6^. To eliminate the environmental effects, the plants were propagated under the same conditions and their seeds were gathered and planted in the Iranian Biological Resource Center^6^. Based on their artemisinin content and morphology of the glandular secretory trichome (assessing density and area of glandular trichome using fluorescent microscopy and scanning electron microscopy, respectively) at the flowering stage, five *Artemisia* species, including *A*. *annua* L. found in Iran (S1) as a control species, *A*. *khorassanica* Podlech (S2, no artemisinin content and very low glandular trichome density), *A*. *persica* Boiss (S3, low artemisinin content, medium density and low area of glandular trichome), *A*. *deserti* Krasch (S4, high artemisinin content, high density and area of glandular trichome) and *A*. *marschalliana* Sprengel (S5, medium artemisinin content, high density and low area of glandular trichome)^6^ were selected. In our previous study, these five species were evaluated in terms of the expression of artemisinin biosynthesis genes and two other genes (*Aa*-*TTG1* and *Aa*-*TFAR1*) that are involved in trichome formation^6^. It is noteworthy that based on scanning electron micrograph, S2 with very low glandular trichome density and very high non-glandular trichome density and S1 with high glandular trichome density and very low non-glandular trichome density (Supplementary Fig. S2) were included in the experiment. In the current study, in order to have a better insight into artemisinin biosynthesis in species other than *A*. *annua*, these five species (Fig. 3) were evaluated in terms of six terpene synthase (*Aa*-*ECS*, *Aa*-*CPS*, *Aa*-*GAS*, *Aa*-*BFS*, *Aa*-*ADS*, and *Aa*-*SQS*, Fig. 1-b), three transcription factor (*Aa*-*ORA*, *Aa*-*ERF1*, *Aa*-*WIRKY1*) and two transporter (*Aa*-*ABCG6* and *Aa*-*ABCG7*, Fig. 1-b) genes expression. In addition, to determine the relationship between ABCG transporter gene expression with artemisinin content and glandular trichome density, these traits were re-evaluated. The transcription levels of the above-mentioned genes of four species including S2, S3, S4 and S5 were relatively compared to S1, which was chosen as a reference species. Half of each leaf (upper branches) was cut and mixed for RNA extraction and expression analysis, and another half was considered for artemisinin measurement at flowering stage. Artemisinin and RNA extraction were performed with three replications and each replication was a mixture of three sampled plants (upper branches). Since the glandular trichome area of these five species varied, chromosome number and nuclear DNA content were also determined employing flow cytometry in order to distinguish the ploidy level of species, as well as find out any possible relationship between gland size and monoploid genome size.

### Fluorescence microscopy

Glandular trichome density of the abaxial leaf epidermis (upper branches) was evaluated. Leaf samples were analyzed under the Olympus IX-71 Inverted Fluorescence Microscope (Olympus, Tokyo, Japan) for assessment of glandular trichome density. All tissue images were taken using the same magnification (10x objective, 10x ocular, Fig. 3). Each replication was the average of three samples.

### Artemisinin extraction

For HPLC (high performance liquid chromatography) analysis, the leaves were sampled from the upper branches and dried in the dark. Artemisinin was extracted from the leaves employing the procedure described by Peng *et al*.^49^. The artemisinin content of the extracts was evaluated by an HPLC system (Waters, USA) equipped with a C18 column (NUCLEODUR 100-5 C18 ec, 250 mm × 4.6 mm, China) and detection was conducted at 210 nm wavelength. The acetonitrile: water 65: 35% (v/v) was used as a mobile phase with 1 ml/min flow rate^50^. The retention time of artemisinin reference standard and artemisinin of *Artemisia* species was 8.35 ± 0.05 min. Artemisinin production in *A*. *persica* (S3), *A*. *deserti* (S4) and *A*. *marschalliana* (S5) was verified using spike artemisinin standard in extraction of these species. The calibration curve was constructed by plotting the peak area (y) against concentration (150, 300, 600, 1200, and 2400 ppm) of standard solutions (x). The determination coefficient (R^2^) was 0.9984. The contents of artemisinin (mg g^−1^ DW) were determined employing calibration curves.

### Real-time RT-PCR

Total RNA was extracted using RiboEx Total RNA reagent (GeneAll Biotechnology Co., Ltd., Songpa-gu, South Korea) according to the manufacturer’s instruction. In addition, the extracted RNA was treated with Qiagen RNase-free DNase (Qiagen, 79254, Qiagen Inc., Midland, ON, Canada) according to the manufacture’s instruction to remove any genomic DNA contamination. To ensure non-amplification of possible contaminated genomic DNA, two strategies were used: (1) Conduction of PCR with RNA template for each primer pairs, (2) SQS primer pairs was designed for spanning of an exon-exon junction. The quality and quantity of RNA were evaluated using agarose gel electrophoresis (Supplementary Fig. S3) and Nanodrop (Thermo Scientific, Germany) spectrophotometer analyses, respectively. cDNA was synthesized with 1 µg total RNA using Thermo Scientific Revert-Aid™ First-Strand cDNA Synthesis Kit (Fermentas, K1622, Thermo Fisher Scientific, Hudson, NH, USA) according to the manufacturer’s protocol in order to obtain a 20 µl cDNA solution. The qPCR primers were designed employing Oligo 7 primer analysis software and then checked with Oligoanalyzer tool (eu.idtdna.com/calc/analyzer) and NCBI/Primer-BLAST (www.ncbi.nlm.nih.gov/tools/primer-blast/index.cgi?LINK_LOC=BlastHome). Based on the studies of Olofsson *et al*.^24^ and Salehi *et al*.^6^ *Aa*-*β*-*actin* and *Aa*-*CPR* were selected as reference genes (Table 2). In the first step, mRNA, complete cds of five artemisinin biosynthesis genes of *A*. *deserti* (S4, species with high artemisinin content) were isolated and sequenced. The sequences of these genes were identical to mRNA of artemisinin biosynthesis genes in *A*. *annua* (Supplementary Fig. S4). Also, PCR products of *CPR*, *GAS*, *CPS*, *ORA*, *ABCG6* and *ABCG7* primer pairs and mRNA, partial cds of *Actin* of four studied species were isolated and sequenced. The sequencing showed that these sequences in the four species were identical to those in *A*. *annua* (Supplementary Fig. S5). The qPCR was performed using specific primers (Table 2) on a BioRad MiniOption real-time PCR detection system (Applied Biosystems, Foster City, CA, USA) with the fluorescent dye SYBR®Green Master Mix 2X (Ampliqon, A323402, Denmark) according to the manufacturer’s instructions. 1 μL of the first strand cDNA was used as a template in 20 μL reactions, including 10 μL SYBR®Green PCR Master Mix and three pmol of each primer. The qPCR was run at 95 °C (15 min); 40 cycles at 95 °C (20 s), 57 °C (30 s), 72 °C (30 s) followed by gradient, 60–95 °C (5 s). The dissociation stage was completed to detect possible primer dimers or non-specific products. The qPCR was carried out with three biological replications for each sample and three technical replications for each biological sample. The negative control of the Master Mix in addition to the primers was performed in all qPCR running. The fluorescence data showed good specificity of PCR products [the amplification curve of each primer pairs was sigmoidal in shape and the melting curve showed only one peak that is related to the specific product (after conducting the PCR, specific identity of each amplicon was verified by gel electrophoretic analysis) and there were no primer dimer and non-specific products, Supplementary Fig. S6]. It was remarkable that ct (cycle threshold) of *Actin* and *CPR* in the five *Artemisia* species in this experiment ranged between 21 and 23, and this range was stable in the vegetative and flowering stages. The melting curves of amplicons (Supplementary Fig. S7) and gel electrophoretic analysis (Supplementary Fig. S8) verified specific amplifications of *Actin* and *CPS* primers pairs in the five *Artemisia* species. Efficiencies of all primer pairs were computed with cDNA serial dilutions using this formula: E = 10^−1/slope^ − 1. The efficiency of all primer pairs ranged between 0.973 and 0.995. Relative expression levels were calculated using the 2^−∆∆CT^ method^51,52^.

**Table 2:** Primer nucleotide sequences used in qPCR.

Genes	accession number (Gene Bank)	Forward and Reverse Primer Sequences	Fragment size (bp)
*Aa*-*β*-*actin*	EU531837	F: 5′-CCCCTGCTATGTATGTTGCCA-3′	202
		R: 5′-CGCTCGGTAAGGATCTTCATCA-3′	
*Aa*-*CPR*	EF197890	F: 5′-CGGAACAGCCATCTTATTCTTCG-3′	149
		R: 5′-GTTGCACGTACTCCTTAGTGG-3′	
*Aa*-*ECS*	AJ001539	F: 5′-GCAACAAGCCTACGAATCACTCAA-3′	126
		R: 5′-CGTGAAAAATTAAGGACCCTCATAGC-3	
*Aa*-*CPS*	AF472361	F: 5′-GAGGCGACATATTTGAGAGTGC-3′	116
		R: 5′-GATAGTGTTGGGTTGGTGTGA-3′	
*Aa*-*GAS*	DQ447636	F: 5′-CAAAGTGGTGGAAGGATATGAGGT-3′	202
		R: 5′-AGGCGAATCTCTTCAATGGTAGC-3′	
*Aa*-*BFS*	AY835398	F: 5′-CAAGGAGGAACAAGAGAGAGG-3′	176
		R: 5′-GCATAAGTAGAGGAAATGGGACA-3′	
*Aa*-*SQS*	AY445505	F: 5′-TGAGGTTTTCAGGGGTGTAGTC-3′	166
		R: 5′-CCTAGTGATGGTCGTTTGGGCA-3′	
*Aa*-*ADS*	HQ315833	F: 5′-CCGAGCAAGAAAGAAAACATAG-3′	203
		R: 5′-AACTTCAAGAAACTGGCACA-3′	
*Aa*-*ORA*	JQ797708	F: 5′-GGCGAGATTATGGCTTGGTACG-3′	184
		R: 5′-CGATGGTTGATGTGGTTCTTGTG-3′	
*Aa*-*ERF1*	JN162091	F: 5′-TGAACTTCCCACATAGAATCGG-3′	148
		R: 5′-TCAACTACCTCAGCCAATGATAC-3′	
*Aa*-*WIRKY*	FJ390842	F: 5′-CAAGAACTACCAAGACCGAATCC-3′	210
		R: 5′-GGAGATAACAGGTGGCGAATAGAC-3′	
*Aa*-*ABCG6*	Aan.67737	F: 5′-CGATAGCCAATAGCCATAAGTG-3′	195
		R: 5′-ATCCTACATTGCTTTCCATACG-3′	
*Aa*-*ABCG7*	Aan.68336	F: 5′-GGTATCTGTAAATGGGGCAAAGTC-3′	173
		R: 5′-ACAATGGCATCCTCAACAACAC-3′	

### Cytogenetic studies

#### Chromosome analysis

Growing roots were used for cytogenetic studies. The best technique for mitosis study is the use of root tip meristem tissues for preparation of karyotype. The appropriate root length and time (when the largest number of cells are in metaphase) were chosen to cut the roots. For the cytological preparations, about 1 cm long growing roots were isolated and pretreated in 0.02% (w/v) colchicine for 3.5 h at room temperature (RT) in the dark to induce metaphase arrest, followed by washing (each for 5 min) in dsH_2_O three times (each for 5 min) and immersion in freshly prepared 3: 1 (v/v) absolute ethanol: glacial acetic acid for 24 h at 4 °C. The fixed roots were hydrolyzed in 5 M HC1 for 10 min at RT, then washed (each for 5 min) in dsH_2_O three times and stained in 2% (w/v) aceto-orcein for 3 h at RT. The five well-spread monolayer metaphase plates from different individuals were analyzed for each *Artemisia* species. High resolution microscopic digital photographs were taken employing an Olympus BX50 microscope (Olympus Optical Co., Tokyo, Japan) equipped with an Olympus DP12 digital camera (Olympus Optical Co., Tokyo, Japan). Six chromosomal parameters including long arm length (L), short arm length (S), chromosome length (CL), arm ratio (AR; L/S), total chromosome volume (TCV = πr^2^ CL) where r = mean chromosome radius and centromeric index (CI = S/CL) were estimated (Supplementary Table S1). Stebbins asymmetry categories^53^ were also identified (Supplementary Table S1).

#### Flow cytometric (FCM) analysis

FCM studies were conducted using propidium iodide (PI) staining technique and *Pisum sativum* cv. Ctirad (2C DNA = 9.09 pg^54^) as an internal reference standard plant. 1 cm^2^ of plant materials (leaves of *Artemisia* species and *Pisum sativum* cv. Ctirad) were chopped in a petri dish with a sharp razor blade in 1 ml of woody plant buffer (WPB^55^) followed by filtering of the nuclei suspension using a Partec (Partec, Münster, Germany) 30 µm nylon mesh. Then, 50 µl of PI and 50 µl of RNase were added to the nuclei suspension. To determine the amount of genomic 2C DNA, the nuclei suspension was analyzed by BD FACSCantoTM-KE flow cytometer (BD Biosciences, Bedford, MA, USA) equipped with an argon ion laser (488 nm) using BD FACSDiva^TM^ software. Three replications were considered for genome size measurements. Histograms were gated employing Partec (Partec, Münster, Germany) FloMax ver. 2.4e. The measurements of relative fluorescence intensity of stained nuclei were performed on a linear scale. The amount of absolute DNA of a sample was computed based on the values of the G1 peak means^31,54^ as follows: Sample 2C DNA (pg) content = (sample G1 peak mean/standard G_1_ peak mean) × standard 2C DNA amount (pg). 1Cx-DNA value was calculated based on a conversion formula proposed by Doležel *et al*.^31^; where 1 pg of DNA represents 978 mega base pairs (Mbp).

### Statistical analysis

The experiment was carried out using a completely randomized design (CRD) with five replications for karyological data and three replications for nuclear DNA content, artemisinin content and gene expression. After initially testing the normal distribution of the data, analyses of variances were conducted using PROC GLM of SAS^56^. Mean comparisons were done by Fisher’s least significant differences (LSD) at 0.01 probability level. In addition, the standard error (SE) was computed.

## Electronic supplementary material

Supplementary Material
