# Author Correction: Expression of key genes affecting artemisinin content in five *Artemisia* species

**DOI:** 10.1038/s41598-018-32883-4

**Published:** 2018-10-15

**Authors:** Maryam Salehi, Ghasem Karimzadeh, Mohammad Reza Naghavi, Hassanali Naghdi Badi, Sajad Rashidi Monfared

**Affiliations:** 1 Department of Plant Genetics and Breeding, Faculty of Agriculture, Tarbiat Modares University, Tehran, P. O. Box 14115-336 Iran; 2 Agronomy and Plant Breeding Department, Agricultural College, University of Tehran, Karaj, Iran; 3 Medicinal Plants Research Center, Institute of Medicinal Plants, ACECR, Karaj, Iran; 4 Department of Agricultural Biotechnology, Faculty of Agriculture, Tarbiat Modares University, Tehran, Iran

Correction to: *Scientific Reports* 10.1038/s41598-018-31079-0, published online 23 August 2018

The original version of this Article contained an error in the affiliation of Hassanali Naghdi Badi, which was incorrectly given as ‘Cultivation and Development Department of Medicinal Plants Research Center, Institute of Medicinal Plants, ACECR, Karaj, Iran’. The correct affiliation is listed below:

Medicinal Plants Research Center, Institute of Medicinal Plants, ACECR, Karaj, Iran.

This error has now been corrected in the HTML and PDF versions of the Article

