# Supplementary material for: Expression of key genes affecting artemisinin content in five Artemisia species
Source: Sci Rep. 2018 Aug 23;8:12659. doi: 10.1038/s41598-018-31079-0 (PMC6107673; doi:10.1038/s41598-018-31079-0)
Supplement: Supplementary file 1 — Supplementary Material [file 41598_2018_31079_MOESM1_ESM.docx]

**Title:** Expression of key genes affecting artemisinin content in five *Artemisia* species

**Authors:** Maryam Salehi, Ghasem Karimzadeh, Mohammad Reza Naghavi, Hassanali Naghdi Badi and Sajad Rashidi Monfared

**Supplementary Information 1**

**Fig. S1.** Scanning electron micrographs of leaves of five *Artemisia* species including *A. annua* found in Iran (S1, Area = 1050 μm^2^ ± 132.3), *A. persica* (S3, Area = 297 μm^2^ ± 25.2), *A. deserti* (S4, Area = 3630 μm^2^ ± 206.6), and *A. marschalliana* (S5, Area = 320 μm^2^ ± 26.8)*.* S1, S4, S5 (× 500, Scale bar 100 μm), S3 (× 2000, Scale bar 10 μm, Salehi et al. 2018).


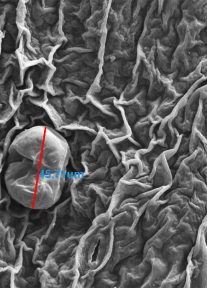

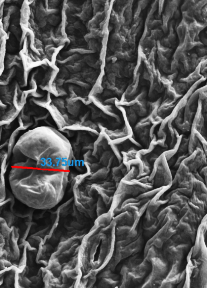


S1


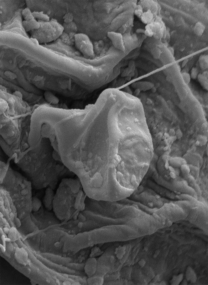

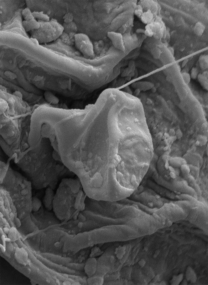


S3


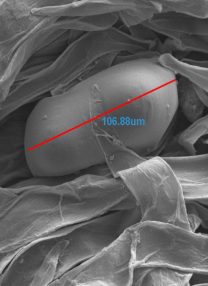

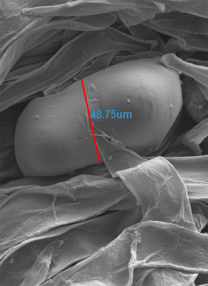


S4


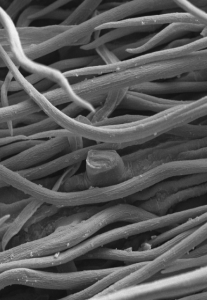

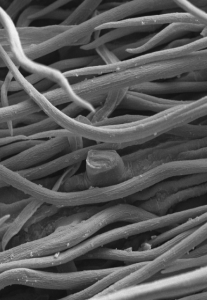


S5

**Supplementary Information 2**

**Fig. S2**. Scanning electron micrographs of leaves of *A. annua* found in Iran (J, K, L) and *A. khorassanica*. (S, T, U); J, S (× 75, Scale bar 100 μm); K, T (× 250, Scale bar 100 μm); L, U (× 500, Scale bar 100 μm, Salehi et al. 2018).


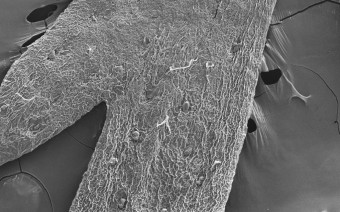

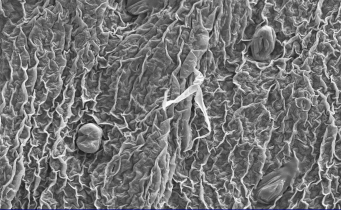

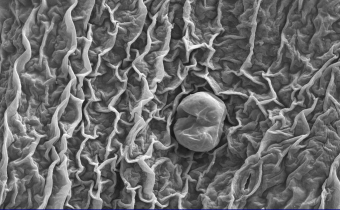


**J**

**K**

**L**


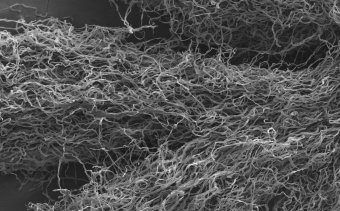


**S**


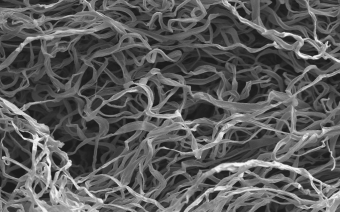


**T**


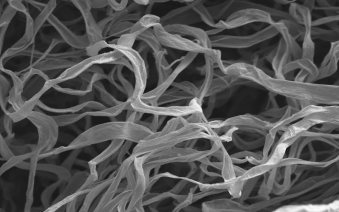


**U**

**Supplementary Information 3**

**Fig. S3.** Agarose gel electrophoresis of RNA extracted from five studied *Artemisia* species.


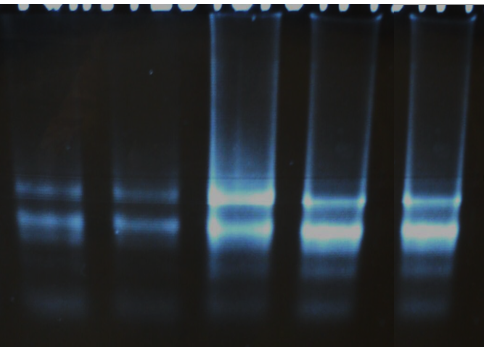


28S rRNA

18S rRNA

5S rRNA

**Supplementary Information 4**

**Fig. S4.** Alignments of the mRNA sequences of artemisinin biosynthesis genes (a, *ADS*; b, *DBR2*; c, *ALDH1*; d, *CYP* and e, *RED1*) from *A. deserti* with *A. annua.*


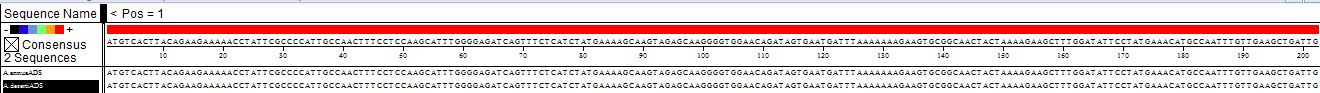

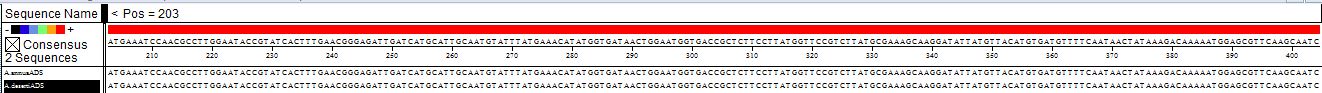

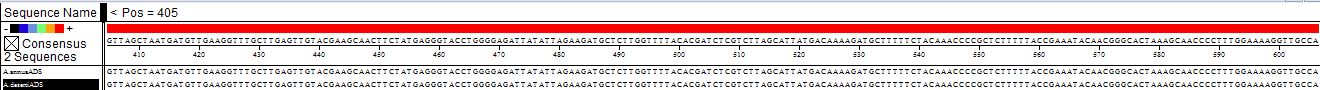

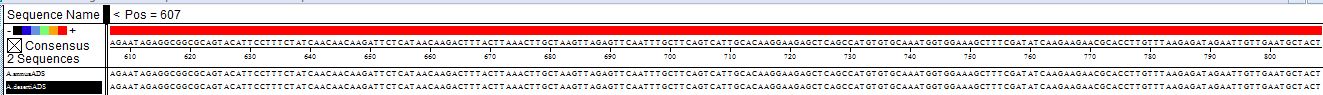

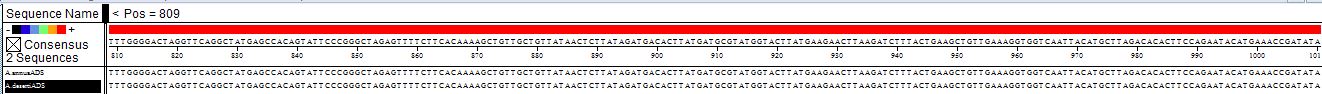

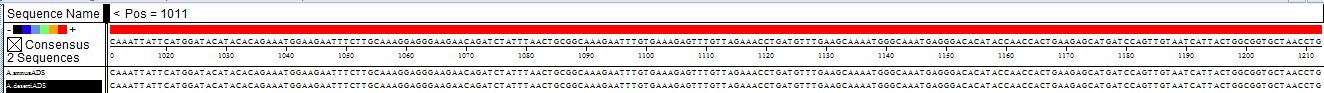

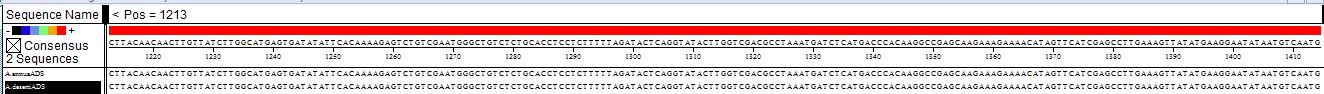

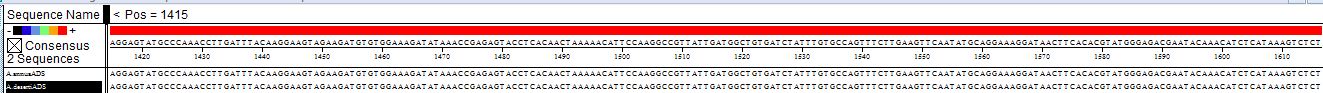

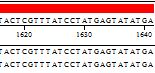


Fig. S4-a.


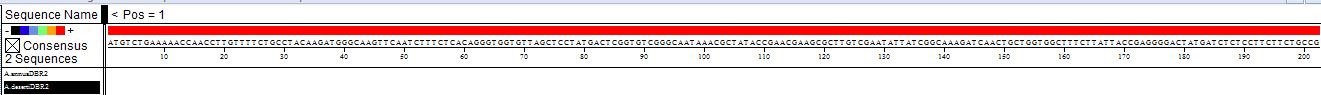

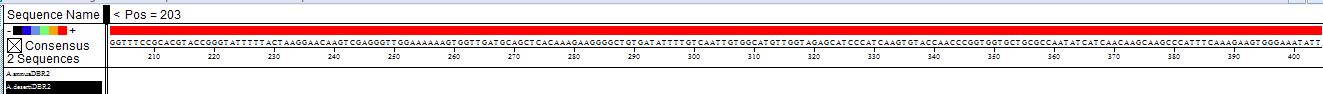

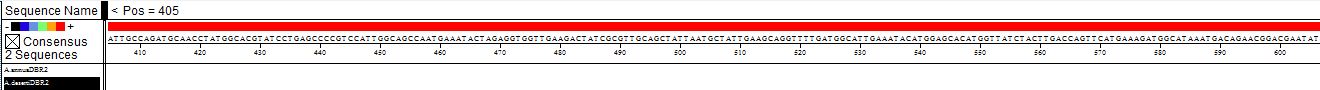

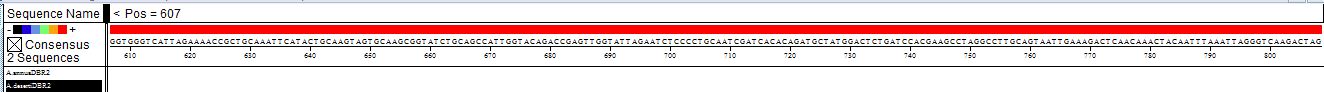

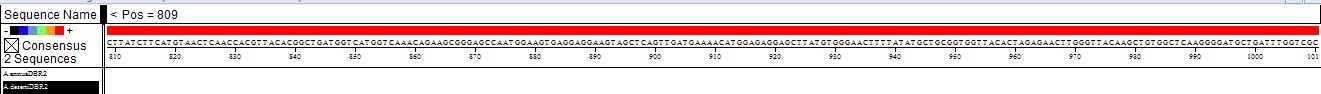

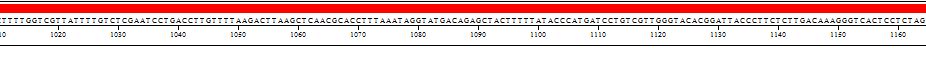


Fig. S4-b.


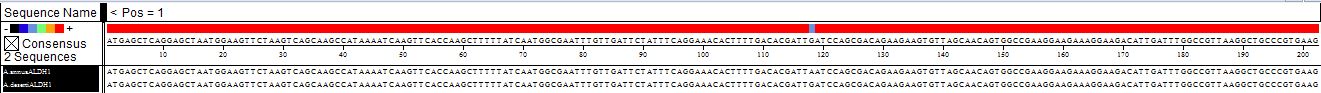

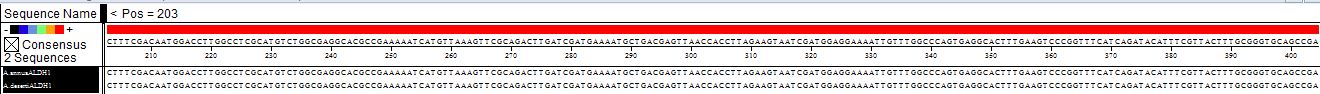

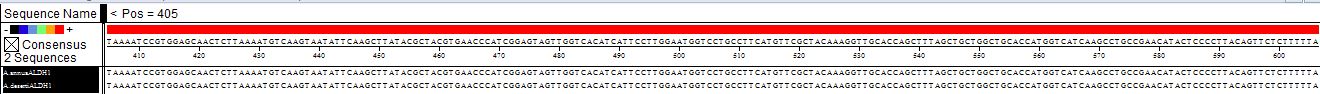

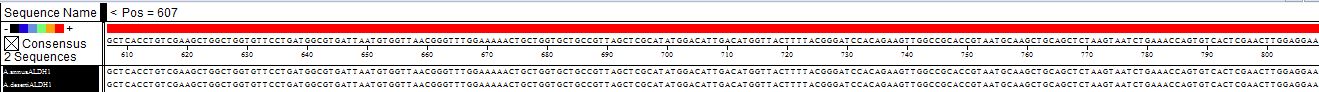

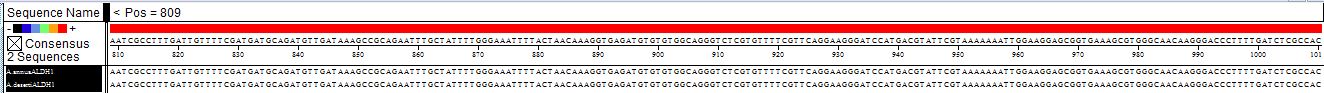

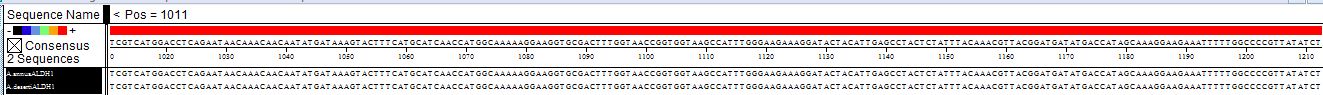

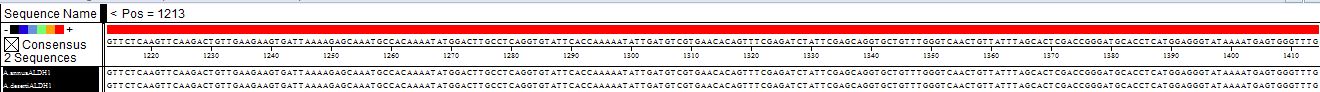

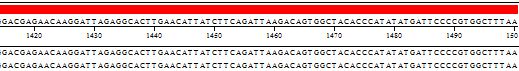


Fig. S4-c.


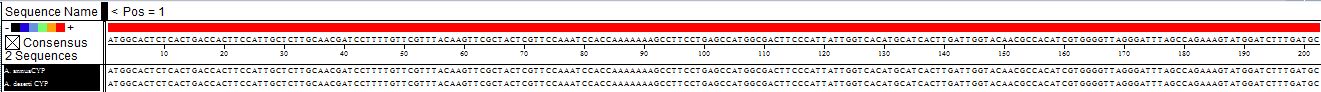

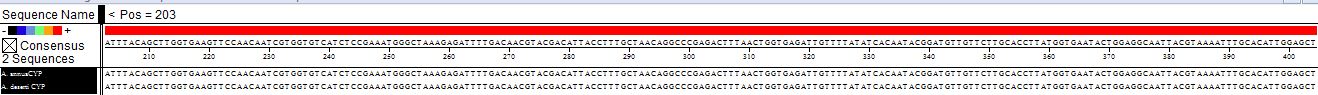

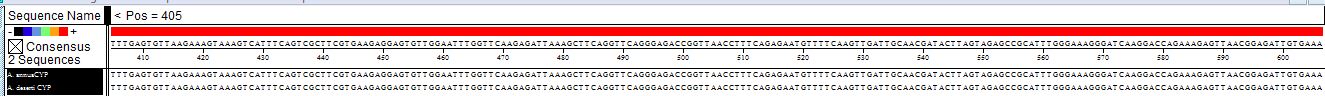

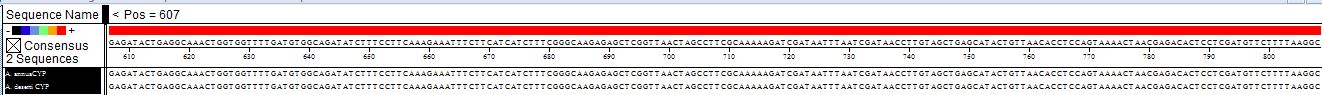

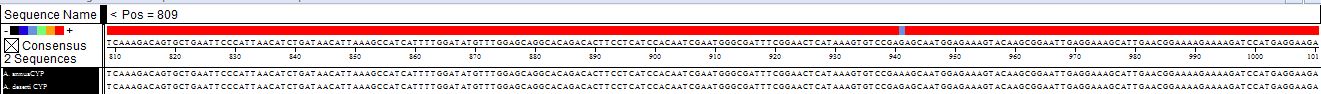

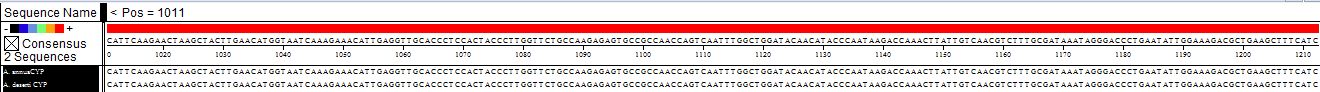

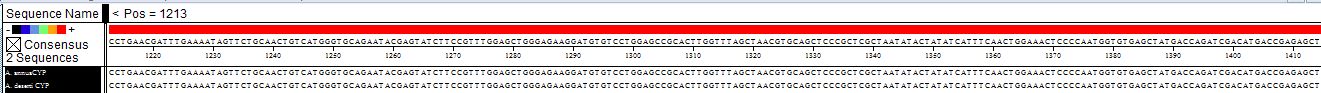

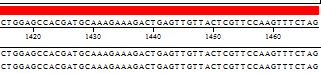


Fig. S4-d.


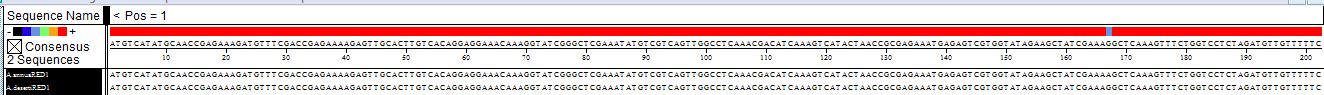

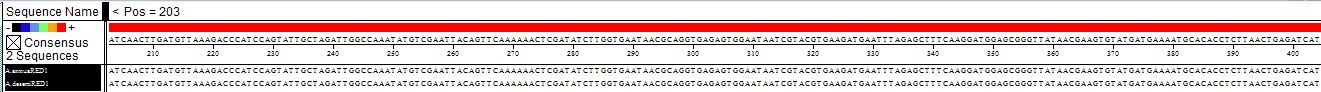

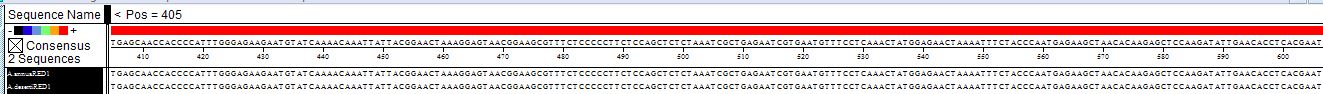

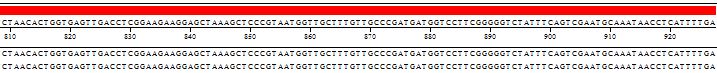

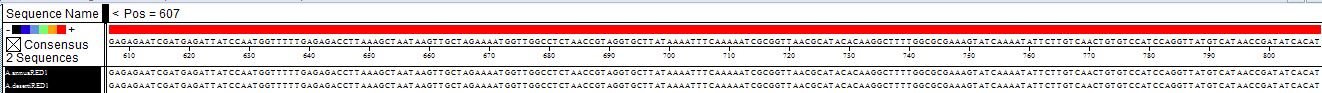


Fig. S4-e.

**Supplementary Information 5**

**Fig. S5.** Alignments of the partial mRNA sequences of *Actin* (a) and PCR products of *CPR* (b), *CPS* (c), *GAS* (d), *BFS* (e), *ORA* (f), *ABCG6* (g) and *ABCG7* (g) primers pairs of five studied *Artemisia* species.


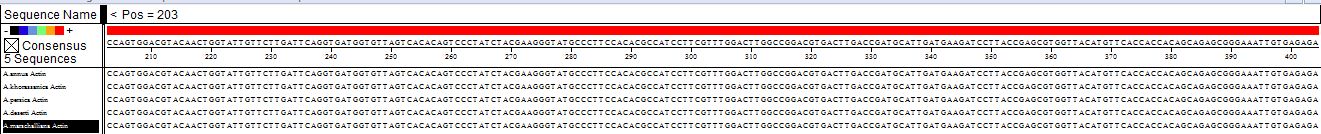

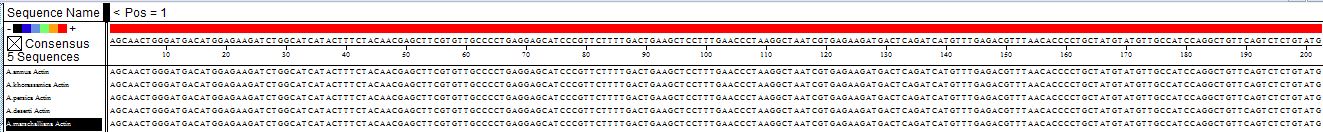

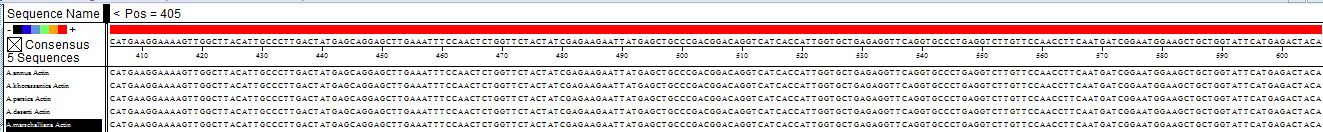

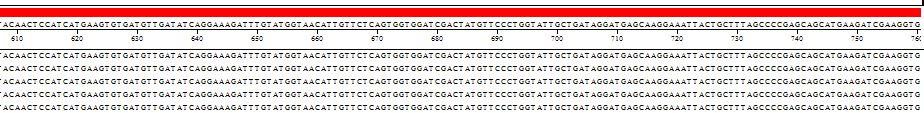


Fig. S5-a.


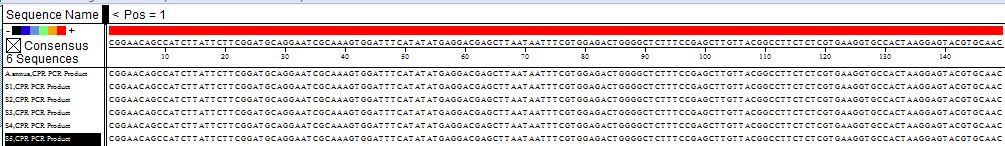


Fig. S5-b.


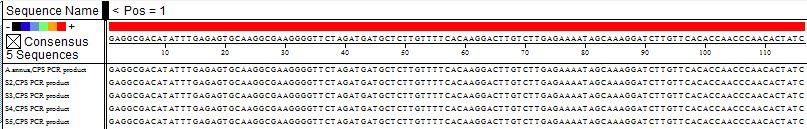


Fig. S5-c.


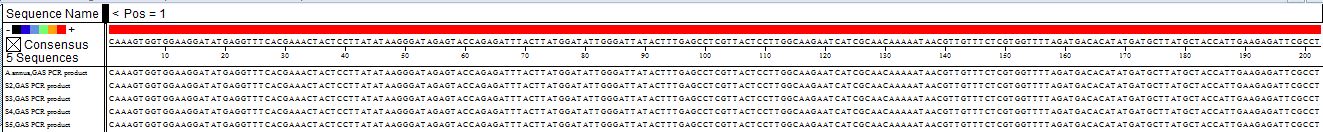


Fig. S5-d.


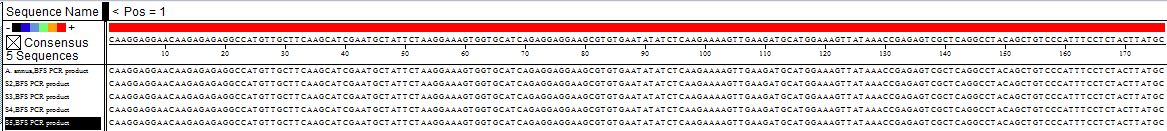


Fig. S5-e.


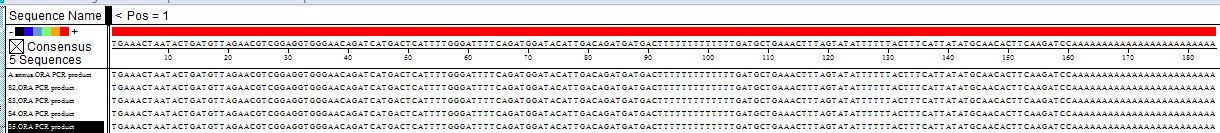


Fig. S5-f.


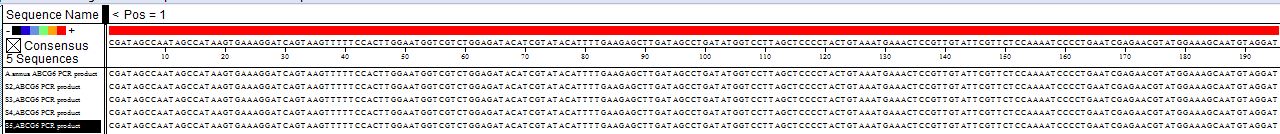


Fig. S5-g.


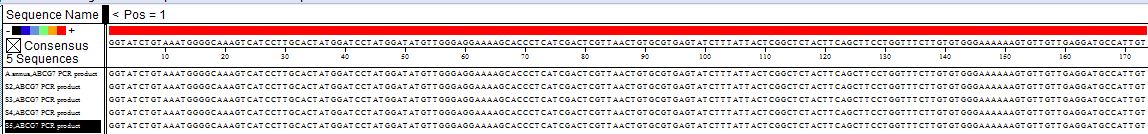


Fig. S5-h.

**Supplementary Information 6**

**Fig. S6.** The amplification (a1, b1, c1, d1, e1, f1, g1, h1, i1, j1, k1, l1, m1, n1, o1, p1, q1, r1) and melting curves (a2, b2, c2, d2, e2, f2, g2, h2, i2, j2, k2, l2, m2, n2, o2, p2, q2, r2) of amplicons of negative control (a) and of studied primers pairs including *β-Actin* (b), *CPR* (c), *ADS* (d), *CYP* (e), *ALDH1* (f), *DBP2* (g), *RED1* (h), *ORA* (i), *ERF1* (j), *WIRKY1* (k), *ABCG6* (l), *ABCG7* (m), *BFS* (n), *GAS* (o), *CPS* (p), *ECS* (q), *SQS* (r).


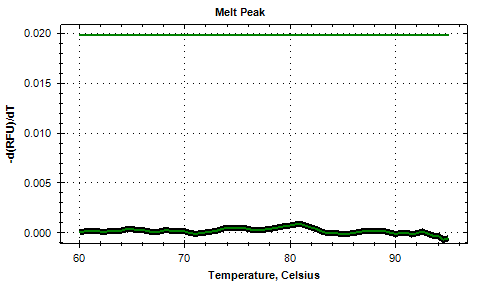

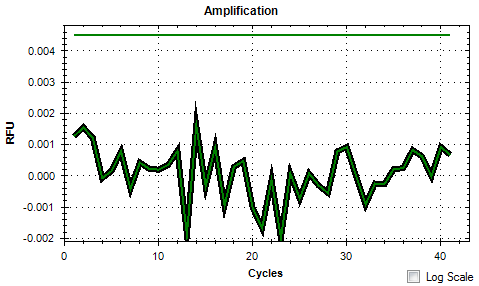

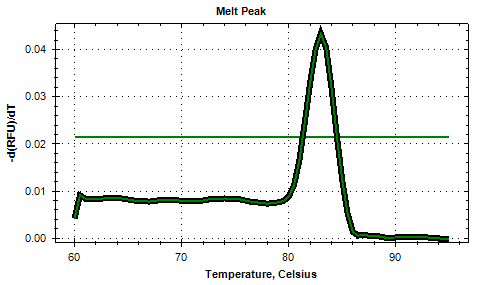

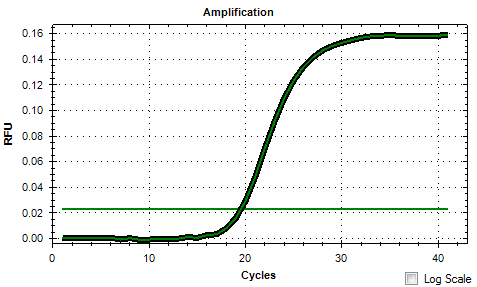

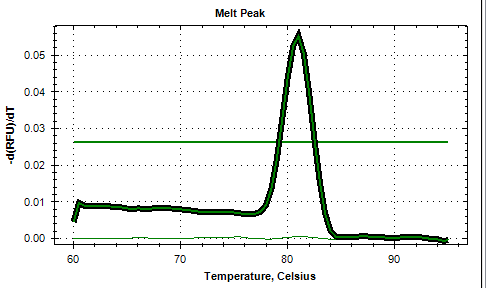

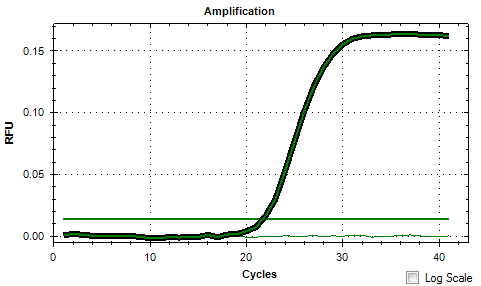

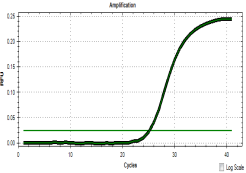

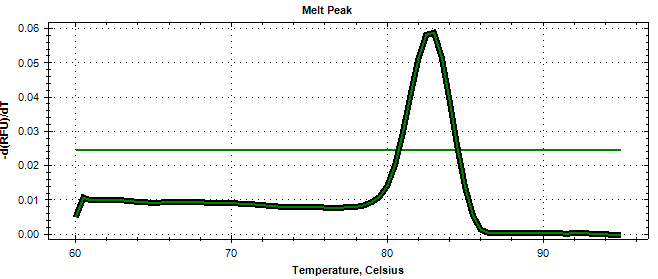

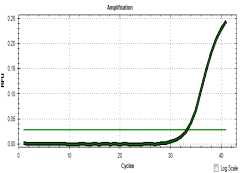

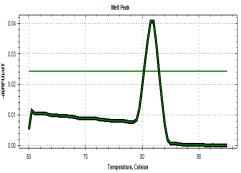

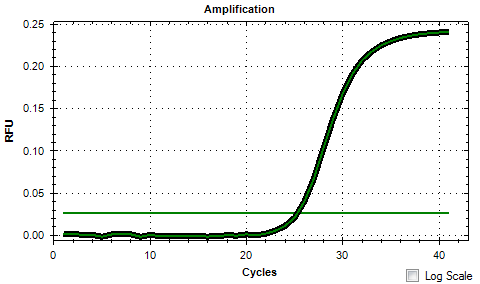

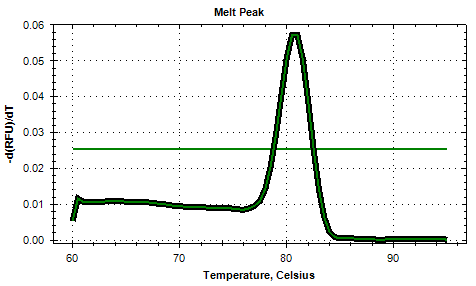

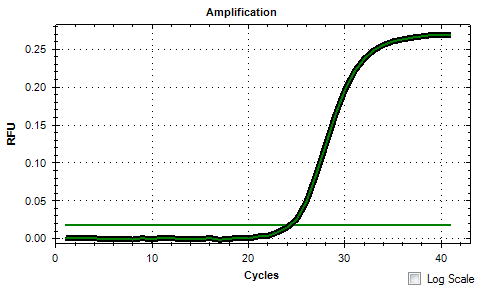

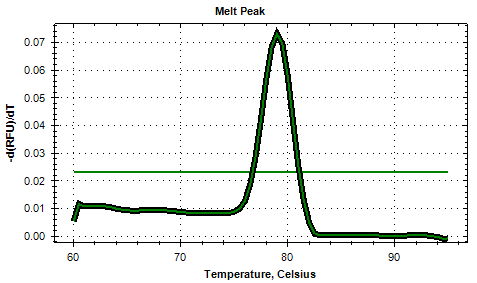

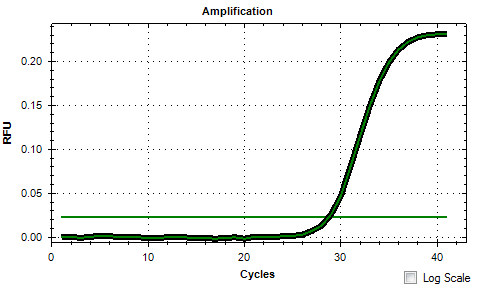

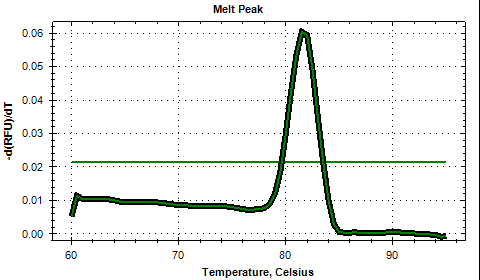

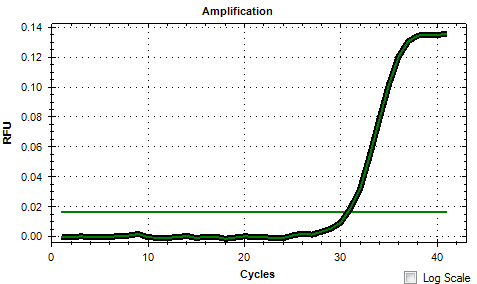

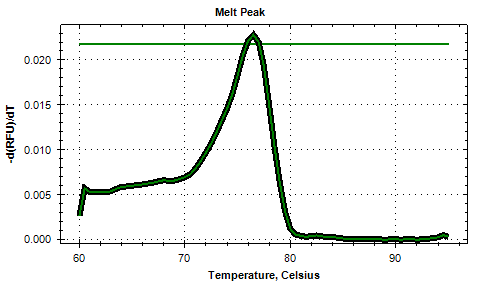

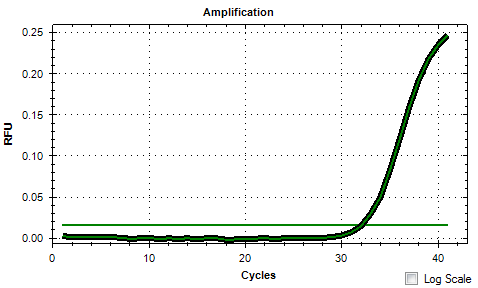

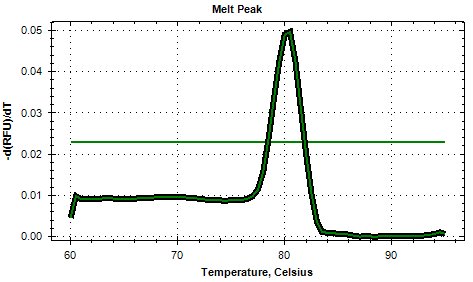

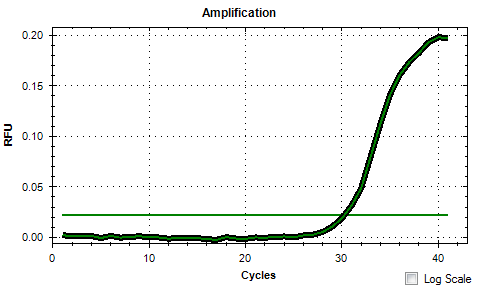

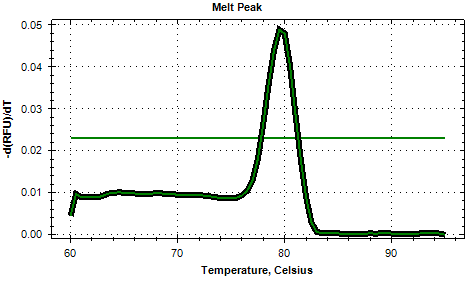

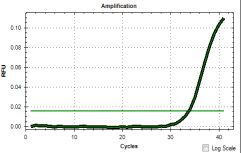

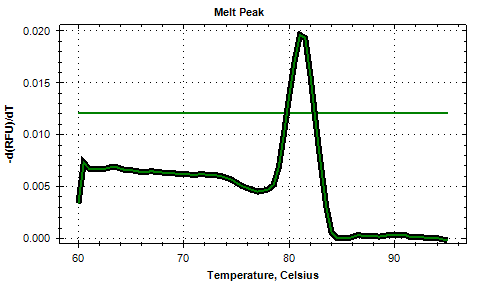

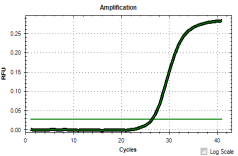

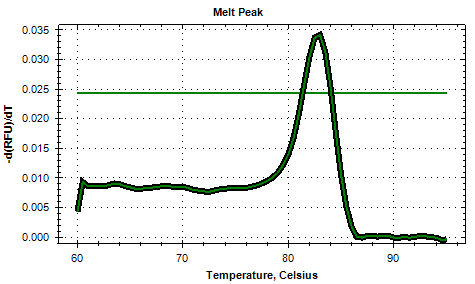

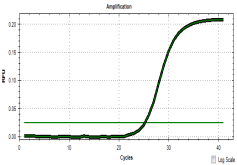

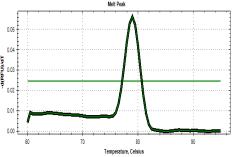

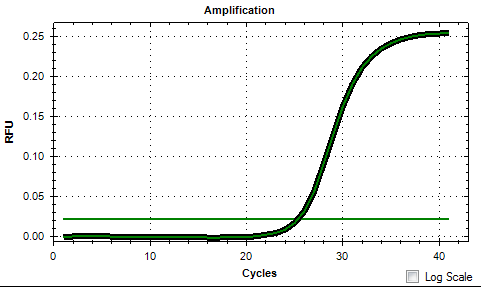

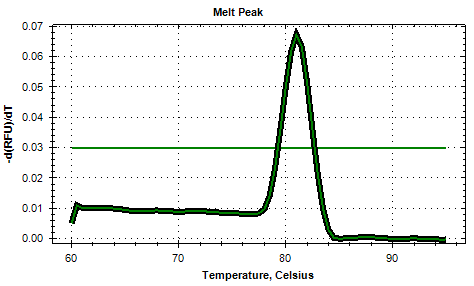

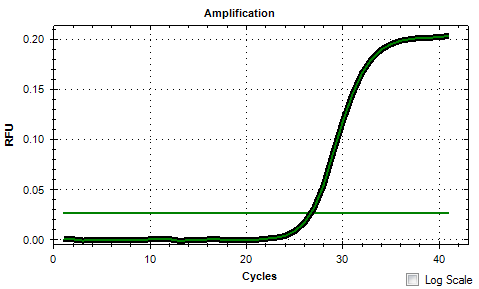

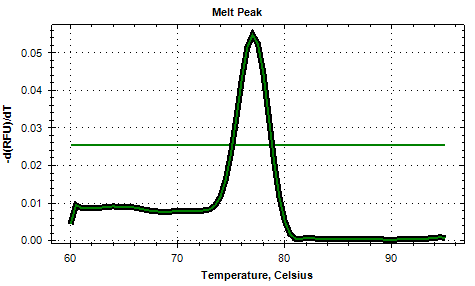

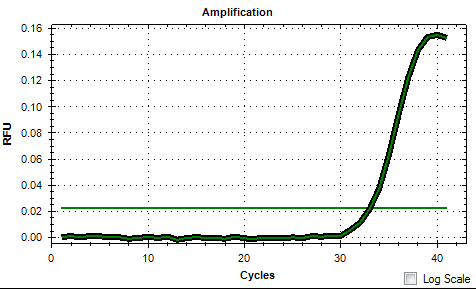

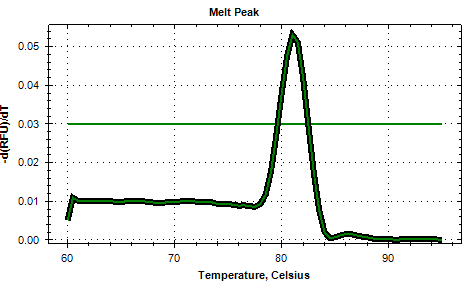

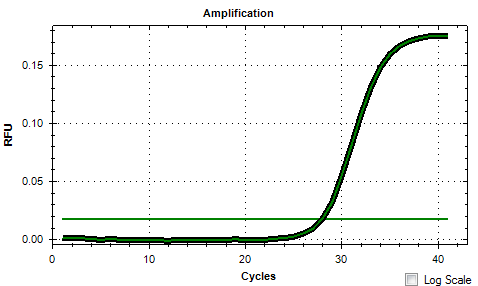

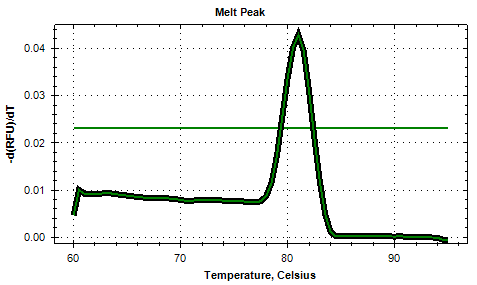


c1

c2

d1

d2

e2

f1

f2

g1

g2

h1

h2

i2

j1

j2

k1

k2

l1

l1

l2

m1

m2

n1

o1

o2

p1

p2

q1

q2

i1

r2

a1

a2

b1

b2

e1

n2

r1

i1

**Supplementary Information 7**

**Fig. S7.** The melting curves of amplicons of *Actin* (a-e) and *CPR* (f-j) primers pairs in five *Artemisia* species including *A. annua* found in Iran (a, f), *A. khorassanica* (b, g), *A. persica* (c, h), *A. deserti* (d, i) and *A. marschalliana* (e, j).


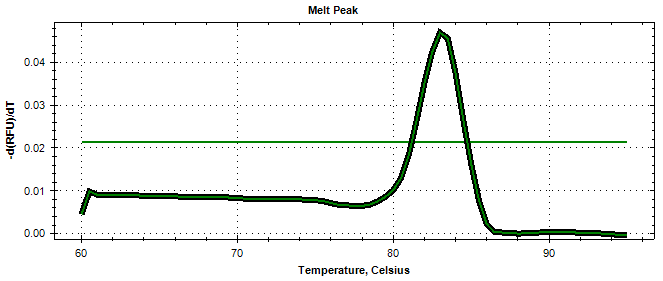


a


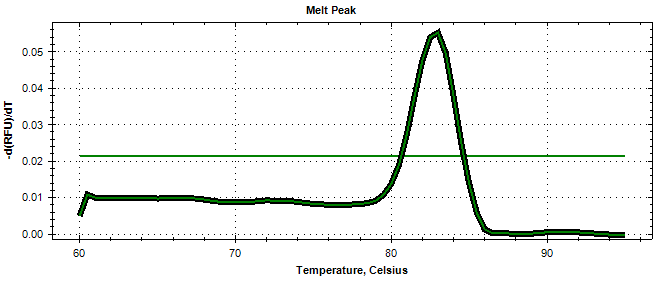


b

c

d

e

f

g

h

i

j

**Supplementary Information 8**

**Fig. S8.** Agarose gel electrophoresis of PCR products of *Actin* (a) and *CPR* (b) primers pairs from five studied *Artemisia* species including *A. annua* (1), *A. khorassanica* (2), *A. persica* (3), *A. deserti* (4), *A. marschalliana* (5), negative control 6). Ma, 100 bp DNA ladder; Mb, 1 KB Plus ladder.

**Supplementary Information 9**

**Table S1.** Chromosomal parameters and some karyotypic symmetry formula of five *Artemisia* species including S1 (*A. annua* found in Iran), S2 (*A. khorassanica*), S3 (*A. persica*), S4 (*A. deserti*), and S5 (*A. marschalliana*)

| Karyotype formula^2^ | Stebbins’ type^1^ | **CI** | **TCV** | **AR** | **CL** | **L** | **S** | **Species codes** |
| --- | --- | --- | --- | --- | --- | --- | --- | --- |
| 18m | 1A | 0.42^b^ ± 0.005 | 4.60^d^ ± 0.10 | 1.39^a^ ± 0.03 | 3.37^d^ ± 0.06 | 1.95^d^ ± 0.04 | 1.42^d^ ± 0.03 | S1 |
| 18m | 1A | 0.44^a^ ± 0.010 | 5.38^c^ ± 0.12 | 1.31^ab^ ± 0.03 | 3.93^c^ ± 0.06 | 2.22^c^ ± 0.04 | 1.72^c^ ± 0.04 | S2 |
| 18m | 1A | 0.42^b^ ± 0.006 | 6.40^b^ ± 0.15 | 1.39^a^ ± 0.04 | 4.67^b^ ± 0.07 | 2.70^b^ ± 0.05 | 1.97^b^ ± 0.04 | S3 |
| 18m | 1A | 0.44^a^ ± 0.004 | 11.76^a^ ± 0.25 | 1.28^b^ ± 0.02 | 6.11^a^ ± 0.12 | 3.41^a^ ± 0.07 | 2.70^a^ ± 0.07 | S4 |
|  |  | 0.531 | 0.299 | 0.531 | 0.315 | 0.184 | 0.176 | LSD_1%_ |
| 36m | 1A | 0.43 ± 0.004 | 5.92 ± 0.08 | 1.34 ± 0.02 | 4.35 ± 0.04 | 2.48 ± 0.03 | 1.88 ± 0.02 | S5 |
| Means within a column followed by the same letter are not significantly different according to the LSD at the 0.01 probability level  ^1^ST: Stebbins classification (1971), ^2^KF: Karyotype formula | | | | | | | | |
